# Systems Medicine of Cancer: Bringing Together Clinical Data and Nonlinear Dynamics of Genetic Networks

**DOI:** 10.1155/2016/7904693

**Published:** 2016-01-11

**Authors:** Konstantin B. Blyuss, Ranjit Manchanda, Jürgen Kurths, Ahmed Alsaedi, Alexey Zaikin

**Affiliations:** ^1^Department of Mathematics, University of Sussex, Falmer, Brighton BN1 9QH, UK; ^2^Barts Cancer Institute, Queen Mary University of London, London EC1M 6BQ, UK; ^3^Potsdam Institute for Climate Impact Research, 14473 Potsdam, Germany; ^4^Department of Mathematics, King AbdulAziz University, Jeddah 21589, Saudi Arabia; ^5^Lobachevsky State University of Nizhny Novgorod, Nizhny Novgorod 603950, Russia; ^6^Institute for Women's Health and Department of Mathematics, University College London, London WC1E 6AU, UK

The last few years have witnessed major developments in experimental techniques for analysis of cancer, including full genome sequencing, measurement of multiple oncomarkers, DNA methylation profile, genomic profile, or transcriptome of the pathological tissue. Whilst these advances have provided some additional understanding of cancer onset and development, the problem of cancer is far from solved. Since the amounts of data emerging from these are very substantial, and the data itself can be extremely heterogeneous (qualitative, quantitative, and verbal descriptions), this makes standard data analysis techniques not practically applicable. One promising direction of addressing the problem of analysis of cancer data is to use modern and sophisticated methods from systems biology and cybernetics. Some challenges of this approach are connecting the results of mathematical analysis with real clinical data, and bridging the existing gaps between the communities of clinicians and applied mathematicians.

This special issue showcases some of the most recent developments in the areas of nonlinear dynamics, mathematical analysis and modeling, data analysis, and simulations in the area of cancer. Having received 11 submissions, six best papers were chosen and published to provide an overview of the research field and to motivate further study.

In “Optimal Placement of Irradiation Sources in the Planning of Radiotherapy: Mathematical Models and Methods of Solving,” O. Blyuss et al. analyse optimal choice of placement of irradiation sources during radiotherapy as an optimization problem. Using the techniques of nondifferentiable optimization and an approximate Klepper's algorithm, the authors derive a new approach for solving this problem and illustrate their methodology with actual numerical simulations of different scenarios.

The paper “Time-Delayed Models of Gene Regulatory Networks” by K. Parmar et al. provide an overview of existing mathematical techniques applicable to modeling the dynamics of gene regulatory networks. The authors focus on the effects of transcriptional and translational time delays and demonstrate how stability of different steady states and the associated behavior change depending on system parameters and the time delays. They contrast the dynamics of the fast mRNA regime, as described by a reduced model, with the dynamics of the full system to illustrate possible differences in behaviour and to highlight the important role played by the time delays.

H. Namazi and M. Kiminezhadmalale in their paper titled “Diagnosis of Lung Cancer by Fractal Analysis of Damaged DNA” discuss how DNA sequences emerging from patient blood plasma can be studied by analysing DNA walks. Comparing the features of DNA profiles for healthy individuals with those for lung cancer patients, the authors derive several predictive criteria for lung cancer based on Hurst exponents and fractal dimension.

The circadian physiology, clock genes, and cell cycle may critically affect results of cancer chronotherapeutics; hence, investigation of gene regulatory networks controlling circadian rhythms is an irreplaceable part of systems medicine of cancer. R. Heussen and D. Whitmore study how the circadian clock is entrained by light, and they experimentally investigate whether there exists a threshold for this synchronization. Moreover, analysis of the constructed numerical model shows that stochastic effects are an essential feature of the circadian clock that provides an explanation of signal decay from the zebrafish cell lines in prolonged darkness.

Some cancer types are especially dangerous because of the high progression rate, and malignant gliomas represent one of the most severe types of tumors. Modern medical approaches offer sophisticated treatment procedures, based on microsurgical tumor removal combined with radio- and chemotherapy. A success of these surgical resection depends on the clarity of intraoperative diagnostics of human gliomas, and the review of O. Tyurikova et al. surveys the wide diversity of modern diagnostic methods used in the course of glial tumor resections.

Recent development in systems biology and medicine have enabled us to analyse and infer networks of interactions and to search for new network oncomarkers. S.-M. Wang et al. in their paper about the identification of dysregulated genes and pathways in clear cell renal cell carcinoma provide an example of this research direction. Using systematic tracking the dysregulated modules of reweighted protein-protein interaction networks they successfully identified dysregulated genes and pathways for this type of cancer, thus gaining insights into possible biological markers or targets for drug development.

We hope that the readers will find the papers published in this special issue interesting, and this will encourage and foster further research on developing new and efficient techniques in systems biology and data analysis for predicting the onset and for monitoring the progression of cancer.

*Konstantin B. Blyuss* *Konstantin B. Blyuss* *Ranjit Manchanda* *Ranjit Manchanda* *Jürgen Kurths* *Jürgen Kurths* *Ahmed Alsaedi* *Ahmed Alsaedi* *Alexey Zaikin* *Alexey Zaikin*

